# Genomic characterization and assessment of the virulence and antibiotic resistance of the novel species *Paenibacillus* sp. strain VT-400, a potentially pathogenic bacterium in the oral cavity of patients with hematological malignancies

**DOI:** 10.1186/s13099-016-0089-1

**Published:** 2016-02-19

**Authors:** George Tetz, Victor Tetz, Maria Vecherkovskaya

**Affiliations:** Institute of Human Microbiology, LLC, 303 5th Avenue, Suite 2012, New York, NY 10016 USA; First State I. P. Pavlov Medical University, Lev Tolstoy Str. 6/8, Saint Petersburg, Russia 197022

**Keywords:** *Paenibacillus* sp., Antibiotic resistance, Nosocomial, Hematological malignancies, Immunocompromised, Pneumonia, Pathogen

## Abstract

**Background:**

*Paenibacillus* sp. strain VT-400, a novel spore-forming bacterium, was isolated from patients with hematological malignancies.

**Methods:**

*Paenibacillus* sp. strain VT-400 was isolated from the saliva of four children with acute lymphoblastic leukemia. The genome was annotated using RAST and the NCBI Prokaryotic Genome Annotation Pipeline to characterize features of antibiotic resistance and virulence factors. Susceptibility to antibiotics was determined by the Kirby–Bauer disc diffusion method. We used a mouse model of pneumonia to study virulence in vivo. Mice were challenged with 7.5 log_10_–9.5 log_10_ CFU, and survival was monitored over 7 days. Bacterial load was measured in the lungs and spleen of surviving mice 48 h post-infection to reveal bacterial invasion and dissemination.

**Results:**

Whole-genome sequencing revealed a large number of virulence factors such as hemolysin D and CD4+ T cell-stimulating antigen. Furthermore, the strain harbors numerous antibiotic resistance genes, including small multidrug resistance proteins, which have never been previously found in the *Paenibacillus* genus. We then compared the presence of antibiotic resistance genes against results from antibiotic susceptibility testing. *Paenibacillus* sp. strain VT-400 was found to be resistant to macrolides such as erythromycin and azithromycin, as well as to chloramphenicol and trimethoprim–sulphamethoxazole. Finally, the isolate caused mortality in mice infected with ≥8.5 log_10_ CFU.

**Conclusions:**

Based on our results and on the available literature, there is yet no strong evidence that shows *Paenibacillus* species as an opportunistic pathogen in immunocompromised patients. However, the presence of spore-forming bacteria with virulence and antibiotic resistance genes in such patients warrants special attention because infections caused by spore-forming bacteria are poorly treatable.

## Background

Acute leukemia accounts for more than 10,000 deaths annually despite improved treatment regimens and novel cytostatic agents [1]. Pneumonia due to opportunistic Gram-positive *Staphylococcus* spp., *Bacillus* spp., and *Enterococcus* spp. is one of the leading causes of morbidity in these patients, as well as in patients with other forms of hematological malignancies, because of treatment-induced immunosuppression [2, 3].

The oral cavity, which hosts more than 700 commensal bacterial species, is the main reservoir of microorganisms that cause aspiration pneumonia [4, 5]. Thus, investigating the oral microbiome is essential to improve therapeutic strategies, especially for patients with hematological malignancies [6]. However, most commensal bacteria are not yet culturable, and molecular techniques based on cloning and sequencing the ribosomal 16S RNA have been used instead to identify species in the human microbiome [7]. Nevertheless, these techniques are prone to false negatives, such as when one bacterial species masks another, and thus underestimate bacterial diversity [8, 9]. In a previous study, we described *Paenibacillus* sp. strain VT-400, a novel spore-forming bacterium isolated from the saliva of patients with acute lymphoblastic leukemia [10]. The strain has never been previously detected in humans.

Notably, spore-forming bacteria are poorly studied, and only a few such bacteria have been described and are associated with the human microbiota [11, 12]. Spores tolerate high temperature, radiation, and noxious chemicals, harbor genes that confer antibiotic resistance, and allow bacteria to survive in unfavorable conditions [13, 14]. Thus, spores contribute significantly to the persistence of infection and the spread of antimicrobial resistance [15]. Indeed, prophylactic treatments like oral rinses are poorly effective against spores, and are thus not sufficiently reduce the bacterial load in the oropharynx, or prevent aspiration pneumonia in at-risk patients, especially those with underlying pathologies such as hematological malignancies [16, 17]. Therefore, identification and characterization of potentially infectious spore-forming microbial species are critical to improve the management or treatment of patients with acute leukemia.

*Paenibacillus* spp. was not known to cause human disease until recent reports implicated *P. alvei*, *P. thiaminolyticus*, and *P. sputi* in respiratory and urinary tract infection, as well as bacteremia in a patient on hemodialysis [18–20]. In this study, we describe *Paenibacillus* sp. strain VT-400, a novel bacterium isolated from the saliva of four children with hematological malignancies, and investigate its potential to cause pneumonia.

## Methods

### Bacterial strain

*Paenibacillus* sp. strain VT-400 was isolated from the saliva of four children with acute lymphoblastic leukemia who were hospitalized at First Pavlov State Medical University, St. Petersburg, Russia. Unless stated otherwise, the isolate was grown on Columbia agar with 5 % sheep blood (BioMerieux, France) and were stored at −80 °C in Columbia broth (BioMerieux) supplemented with 50 % glycerol. The strain was screened for hemolytic activity by cultivation at 37 °C for 48 h on agar plates supplemented with 5 % sheep blood. Clearing and greenish zones around colonies were considered to indicate β- and α-hemolytic activity, respectively. Primary morphological characterization was performed by light microscopy (Axiostar, Zeiss, Germany), and Gram staining was performed using a kit (Merck, Darmstadt, Germany).

To generate inoculum for infecting mice, the strain was grown at 37 °C for 48 h on Columbia agar with 5 % sheep blood. Colonies picked from the plate were then grown for 18 h at 37 °C in 5 mL Columbia broth. Cells were harvested by centrifugation at 3000×*g* for 15 min (Eppendorf 5415 C centrifuge; Eppendorf Geratgebau GmbH, Hamburg, Germany), and suspended in an isotonic phosphate buffer (0.15 mM, pH 7.2). The turbidity of the suspension was adjusted using a McFarland standard.

### Genome annotation and phylogenetic analysis

Whole-genome sequences from isolates of *Paenibacillus* sp. strain VT-400 were aligned using MUSCLE, and phylogenetic trees were constructed based on the Tamura-Nei distance model in PHYML version 3.0, with 1000 bootstrap replicates [21–23]. The most closely related *Paenibacillus* genomes were included in the analysis. The genome was annotated and mined for virulence factors and antibiotic resistance genes using Rapid Annotation using Subsystems Technology (RAST) and the NCBI Prokaryotic Genome Annotation Pipeline [24, 25].

### Antimicrobial susceptibility testing

Susceptibility to antibiotics was determined by the Kirby–Bauer disc diffusion method according to criteria defined by the Clinical and Laboratory Standards Institute [26]. The strain was tested for susceptibility to 30 µg amoxiclav, 10 μg ampicillin, 10 U penicillin, 30 µg vancomycin, 30 μg cefotaxime, 10 μg erythromycin, 15 μg azithromycin, 10 µg gentamicin, 30 µg amikacin, 30 μg kanamycin, 2 μg clindamycin, 30 μg doxycycline, 5 µg ciprofloxacin, 30 μg neomycin, 30 μg chloramphenicol, 30 μg tetracycline (Becton–Dickinson, USA) and 1.25 μg/23.75 μg trimethoprim-sulfamethoxazole (Oxoid, UK).

### Pathogenicity in a mouse infection model

Adult C57BL/6 mice weighing approximately 20 g (Rappolovo, North-West region, Russia) were housed in individual cages in a facility free of known murine pathogens, and were provided feeding ad libitum. Animals were cared for in accordance with National Research Council recommendations, and experiments were executed in accordance with the Guide for the Care and Use of Laboratory Animals [27].

Animals were randomly designated into two groups of eight, which were used to measure overall survival and bacterial load. Mice were then anesthetized with 2 % isoflurane, and orally instilled with bacterial suspension as previously described [28]. Briefly, nares were blocked, and mice aspirated 50 µL *Paenibacillus* sp. strain VT400 into the lungs while being held vertically for 60 s. Mice received a total dose of 7.5 log_10_, 8.5 log_10_, or 9.5 log_10_ CFU/mouse. Control mice were treated with sterile 50 µL phosphate-buffered saline. Overall survival was assessed over 7 days, while bacterial load was measured in the lungs and spleen of surviving mice 48 h post infection.

### Microbiological assessment of infected lung and spleen

Bacterial load in the spleen and lungs was measured 48 h post infection. Briefly, surviving animals in groups designated for this assessment were euthanized by CO_2_ and cervical dislocation. Lungs and spleen were collected and homogenized in 1 mL phosphate-buffered saline. As *Paenibacillus* sp. strain VT-400 was found to be resistant to chloramphenicol and trimethoprim, serial tenfold dilutions of tissue homogenates were plated on Columbia agar with 5 % sheep blood, 5 μg/mL chloramphenicol, and 10 μg/mL trimethoprim (Sigma Chemical Co., St Louis, MO, USA), and cultured at 37 °C. Colonies of spore-forming bacteria were counted after 48 h, and bacterial loads are reported as mean log_10_ CFU/g tissue ± SD. Morphology was characterized by light microscopy (Axiostar, Zeiss), and cells were Gram stained using a kit (Merck).

### Ethical approval and consent

Ethical approval was granted by the First State I. P. Pavlov Medical University Ethics Committee (501/M2013). In accordance with ethical approval, consent to use human biological material was assumed following completion of consent forms.

### Statistics

Survival was compared by Kaplan–Meier analysis log-rank test. Differences in bacterial load were evaluated by one-way analysis of variance in SigmaStat version 2.03 (SPSS, Inc., San Rafael, CA). A *P* value <0.05 was considered significant.

## Results

### Phylogenetic analysis

*Paenibacillus* sp. strain VT 400, which has never been detected in humans before, was isolated for the first time from the saliva of pediatric patients with acute lymphoblastic leukemia. In a previous study, whole-genome sequencing was performed on Illumina HiSeq 2500, with 125-fold average coverage [10]. Assembly generated 116 contigs spanning 6,986,122 bp, with G+C content 45.8 %.

**Fig. 1:**
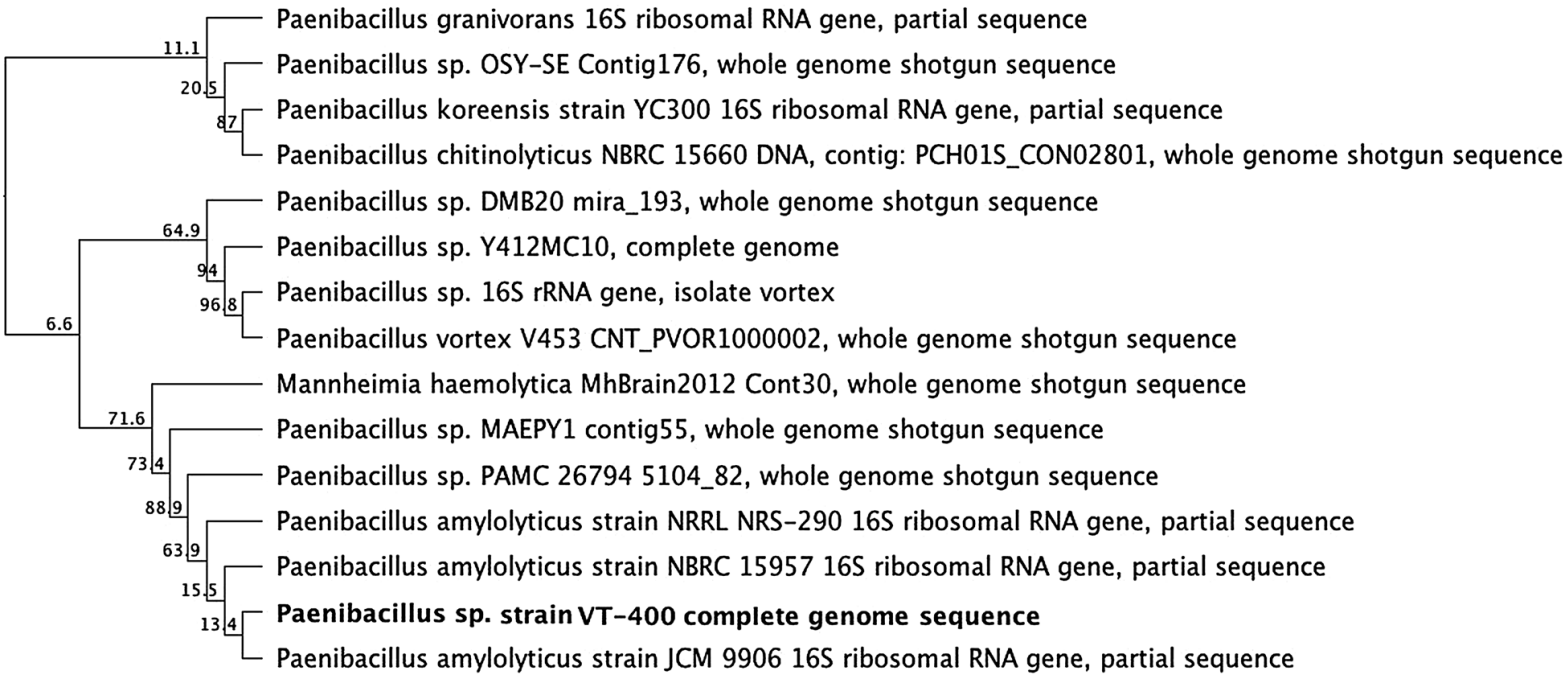
Dendrogram illustrating the relationship of *Paenibacillus* sp. strain VT 400 to the most closely related *Paenibacillus* sequences deposited in GenBank. The tree is based on partial 16S rRNA sequences >1400 bp. Bootstrap proportions in 1000 replicates are shown at branch points

On the basis of these analyses, the strain was identified as a novel species for which *Paenibacillus* sp. strain VT 400 was assigned, and its genome was deposited in GenBank under accession number LELF01000000. Phylogenetic analysis based on 16S rRNA demonstrated that *Paenibacillus* sp. strain VT 400 is clearly distinguished from other species, as well as from other strains of *P. amylolyticus* (Fig. 1).

### Microbiological characteristics of *Paenibacillus* sp. strain VT 400

*Paenibacillus* sp. strain VT 400 is Gram-positive, aerobic, spore-forming, rod-shaped, and motile via peritrichous flagella [10]. Colonies growing on sheep blood agar are smooth, white pearl in color, and from 0.5 to 1 mm in diameter after 24 h at 37 °C in an aerobic atmosphere. β-hemolysis was observed around colonies growing on blood agar plates. The type strain is deposited in the Deutsche Sammlung fur Mikroorganismen und Zellkulturen (Braunschweig, Germany) under accession number DSM 100755.

### Genes encoding virulence factors and in vivo pathogenicity

Analysis of the genome revealed a large number of genes encoding virulence factors that may contribute to pathogenicity (Table 1) [29]. Most are degradative enzymes and adhesins that may facilitate infection, including proteases, phospholipases, ureases, chitinases, and endopeptidases [30]. Significantly, we found chemotaxis proteins that were previously shown to contribute to bacterial virulence [31]. A couple of toxins or putative toxins were also detected, as well as superantigen CD4+ T-cell-stimulating antigen, which causes severe symptoms and septic shock [32].

**Table 1:** Genes encoding virulence factors in *Paenibacillus* sp. strain VT 400

CDS no.	Functional annotation	CDS no.	Functional annotation
*Toxins or putative toxins*			
WP_017689222.1	Hemolysin D	WP_047842244.1	CD4+ T-cell-stimulating antigen
*Degradative enzymes and adhesins*			
WP_047843127.1	Cell adhesion protein	WP_047843815.1	Peptidase M28
WP_047841133.1	Clp protease *ClpX*	WP_047844415.1	Peptidase M15
WP_047841161.1	CAAX protease	WP_047840296.1	Peptidase S9
WP_047841788.1	Zn-dependent protease	WP_047840642.1	Peptidase S41
WP_036605888.1	Lon protease	WP_047840884.1	Peptidase T
WP_047841635.1	ATP-dependent protease	WP_047841004.1	Peptidase C60
WP_047842474.1	*Clp* protease ATPase	WP_047842822.1	Peptidase S8
WP_047842474.1	RIP metalloprotease *RseP*	WP_047843693.1	Peptidase M20
WP_047843793.1	Zinc metalloprotease	WP_047841259.1	Peptidase M4
WP_047843449.1	Alkaline serine protease	WP_047841848.1	Peptidase C15
WP_036611272.1	O-sialoglycoprotein endopeptidase	WP_047844159.1	Peptidase M22
WP_047842657.1	Oligoendopeptidase F	WP_047842036.1	Peptidase A24
WP_047842959.1	Endoglucanase	WP_047842221.1	Peptidase M56
WP_047841916.1	Chitinase	WP_047842221.1	Oligopeptidase *PepB*
WP_047840281.1	Aminopeptidase	WP_047842554.1	Peptidase E
WP_047840267.1	Methionine aminopeptidase	WP_047843428.1	Peptidase M32
WP_047844227.1	Lysophospholipase	WP_047843333.1	Peptidase M29
WP_047843070.1	Phospholipase D	WP_047843711.1	Peptidase M1
WP_047843459.1	5′-Nucleotidase	WP_047843711.1	Peptidase M16
WP_047841534.1	GDSL family lipase	WP_036610857.1	Urease subunit alpha *ureC*
WP_047842732.1	d-alanyl-d-alanine carboxypeptidase	WP_047842024.1	Urease subunit beta *ureB*
*Flagella components*			
WP_036607291.1	Flagellar motor protein *MotA*	WP_047842476.1	Flagellar motor switch protein *FliG*
WP_036607292.1	Flagellar motor protein *MotB*	WP_047842475.1	Flagellar M-ring protein *FliF*
WP_047842487.1	Flagellar biosynthesis protein *FlhA*	WP_047840678.1	Flagellar synthesis anti-sigma-D factor
WP_047842482.1	Flagellar basal body rod protein *FlgG*	WP_047840677.1	Flagellar biosynthesis protein *FlgN*
WP_047843392.1	Flagellar basal body P-ring biosynthesis protein *FlgA*	WP_047840676.1	Flagellar hook protein *FlgK*
WP_047842488.1	Flagellar GTP-binding protein	WP_047840675.1	Flagellar hook protein *FlgL*
WP_047842486.1	Flagellar biosynthesis protein *FlhB*	WP_047840661.1	Flagellar biosynthesis protein *FliS*
WP_047842485.1	Flagellar biosynthesis protein *FliQ*	WP_036609359.1	Flagellar motor switch protein *FliM*
*Chemotaxis*			
WP_047841047.1	Chemotaxis protein *CheY*	WP_036605799.1	Chemotaxis protein *CheC*
WP_047842491.1	Chemotaxis protein *CheA*	WP_036606984.1	Chemotaxis protein *CheR*
WP_025703561.1	Chemotaxis protein *CheW*	WP_017689162.1	Chemotaxis protein *CheD*

**Fig. 2:**
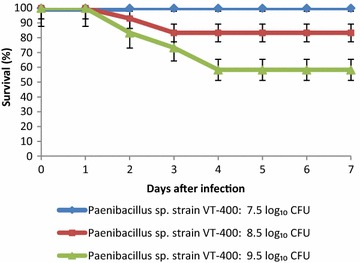
Seven-day survival (%) in mice challenged with 7.5 log_10_, 8.5 log_10_, and 9.5 log_10_ CFU of *Paenibacillus* sp. strain VT 400. *Curves* are representative of three independent experiments, with n = 8 in each treatment

We used a mouse model of pneumonia to study virulence in vivo. Mice were challenged with 7.5 log_10_–9.5 log_10_ CFU, and survival was monitored over 7 days (Fig. 2). All animals exhibited typical signs of acute infection within 24 h, including hypothermia, piloerection, breathing difficulty, narrowed palpebral fissures, trembling, and reduced locomotor activity. There was a direct correlation between severity of symptoms and dose. Accordingly, mortality depended on dose as well, with mortality observed within 48 h in mice exposed to 8.5 log_10_ and 9.5 log_10_ CFU *Paenibacillus* sp. strain VT 400.

Bacterial load was also measured in the lungs and spleen of surviving mice 48 h post infection (Table 2). To confirm the presence of *Paenibacillus* sp. strain VT 400, tissues were homogenized and plated on selective media. Spore-forming bacteria were identified by microscopy. There was approximately 2.47 log_10_ more CFU/g of infected lung tissue in the high-dose group than in the low-dose group (*P* < 0.05). In addition, the data indicated that *Paenibacillus* sp. strain VT 400 spread from the lungs to the spleen, in which bacterial load was also dose-dependent. Taken together, the data suggest that mortality is due to, at least in part, progressive bacterial invasion and dissemination.

**Table 2:** *Paenibacillus* sp. strain VT 400 CFU in the lungs and spleen 48 h post infection

Dose (log_10_ CFU/mouse)	Log_10_ CFU/g tissue, mean ± SD	
	Lung	Spleen
Control	0	0
7.5	0.58 ± 0.28	0.14 ± 0.25
8.5	1.13 ± 0.55	0.25 ± 0.18
9.5	3.05 ± 0.74	1.20 ± 0.34

Moreover, analysis of the *Paenibacillus* sp. strain VT 400 genome revealed an array of proteins involved in or essential for sporulation (Table 3). Phylogenetic analysis indicated that these genes are conserved and are closely related to other members of the *Bacillaceae* family [33].

**Table 3:** Sporulation factors in the *Paenibacillus* sp. strain VT 400 genome

CDS no.	Functional annotation
*Stage 0* (*pre*-*septation*)	
WP_017691423.1	Sporulation protein J
WP_047842196.1	Sporulation protein M
*Stage II* (*post*-*septation*)	
WP_047843799.1	Stage II sporulation protein P
WP_047840704.1	Stage II sporulation protein R
WP_017687629.1	Stage II sporulation protein M
*Stage III* (*engulfment*)	
WP_024632710.1	Stage III sporulation protein D
WP_036674989.1	Stage III sporulation protein AA
WP_036614389.1	Stage III sporulation protein AB
WP_036614387.1	Stage III sporulation protein AE
WP_017687241.1	Sporulation protein YqfC
*Stage IV* (*cortex*)	
WP_036607700.1	Stage IV sporulation protein A
*Stage V* (*spore coat*)	
WP_047844446.1	Stage V sporulation protein AC
WP_047843814.1	Stage V sporulation protein AEB
WP_047843753.1	Stage V sporulation protein D
WP_019424875.1	Stage V sporulation protein M
WP_017689559.1	Stage V sporulation protein S
WP_036606123.1	Stage V sporulation protein T
*Other sporulation proteins*	
WP_036607856.1	Sporulation sigma factor *SigF*
WP_017687309.1	Sporulation sigma factor *SigG*

### Analysis of drug resistance genes and antimicrobial susceptibility testing

Genome analysis also revealed that *Paenibacillus* sp. strain VT 400 harbors different antibiotic resistance genes (Table 4). A total of 96 genes were major facilitator superfamily (MFS) plasma membrane transporters, 18 were multidrug ATP-binding cassette (ABC) transporters [34, 35]. Four genes were identified as multidrug ABC transporter permeases, eight as multidrug and toxic compound extrusion (MatE) transporters, and two as small multidrug resistance (SMR) proteins [36, 37]. A multidrug drug metabolite transporter (DMT) was also detected [38]. Moreover, the *Paenibacillus* sp. strain VT 400 genome also contains genes that confer resistance to specific antibiotics. Finally, genes encoding resistance to tellurium, tunicamycin, and bleomycin were also present. These compounds are used to treat hematological malignancies [39, 40].

**Table 4:** Key antibiotic resistance genes in the *Paenibacillus* sp. strain VT 400 genome

CDS no.	Function	CDS no.	Function
WP_047841924.1 WP_047840904.1	Fosmidomycin resistance protein	WP_047844309.1	Multidrug DMT transporter
WP_047840644.1	Vancomycin resistance protein	WP_047844296.1	Multidrug MFS transporter
WP_047842579.1	Tunicamycin resistance protein	WP_047841225.1	Multidrug ABC transporter ATPase
WP_047840788.1	Bleomycin resistance protein	WP_047840722.1	Multidrug resistance protein SMR
WP_036607427.1	Fosfomycin resistance protein *FosB*	WP_047840931.1	Bacteriocin ABC transporter ATPase
WP_047841800.1	Tellurium resistance protein *TerA*	WP_047844233.1	Multidrug transporter MatE
WP_047840921.1	Tellurium resistance protein *TerF*	WP_047844301.1 WP_047843841.1 WP_047843528.1	Beta-lactamases
WP_026080972.1	Macrolide ABC transporter ATP-binding protein	WP_047842226.1	Metal-dependent hydrolase, beta-lactamase superfamily II
WP_036615192.1	Macrolide transporter	WP_047842143.1	Aminoglycoside phosphotransferase
WP_047840666.1	Cephalosporin hydroxylase	WP_036670493.1	Aminoglycoside adenylyltransferase
WP_047843966.1	MFS transporter	WP_047840993.1	Aminoglycoside 3-N-acetyltransferase
WP_047843373.1	MFS transporter	KLU58081.1	Chloramphenicol acetyltransferase
WP_047843512.1	MFS transporter	WP_047841635.1	Tetracycline resistance protein TetA
WP_047844079.1	Multidrug ABC transporter permease	WP_036614110.1	d-alanine-d-alanine ligase
WP_047844020.1	Multidrug ABC transporter ATP-binding protein	WP_047843376.1	Dihydrofolate reductase

The antibiotic susceptibility of *Paenibacillus* sp. strain VT 400 was then tested against an array of antimicrobials commonly used to treat nosocomial pneumonia [41]. As can be seen from Table 5, the strain was resistant to macrolides such as erythromycin and azithromycin, as well as to chloramphenicol and trimethoprim-sulfamethoxazole. However, it was sensitive to β-lactams, aminoglycosides, glycopeptides, tetracyclines, lincosamides, and fluoroquinolones.

**Table 5:** Antibiotic susceptibility of *Paenibacillus* sp. strain VT 400

Antibiotic	Susceptibility
Amoxiclav	S
Ampicillin	S
Penicillin	S
Vancomycin	S
Cefotaxime	S
Erythromycin	R
Chloramphenicol	R
Azithromycin	R
Gentamicin	S
Amikacin	S
Kanamycin	S
Clindamycin	S
Doxycycline	S
Ciprofloxacin	S
Neomycin	S
Tetracycline	S
Trimethoprim-sulfamethoxazole	R

*S* sensitive, *R* resistant

## Discussion

Bacteria that colonize the oral cavity are important pathogenic agents of pneumonia and other opportunistic infections, especially in immunocompromised hosts. We have now identified one such bacterium, *Paenibacillus* sp. strain VT 400, a novel species that was isolated from children with acute leukemia [28].

Whole-genome analysis indicated that this spore-forming bacterium harbors known virulence factors such as hemolysin, degradative enzymes, adhesins, and flagella. Moreover, CD4+ T-cell-stimulating antigen, a superantigen that causes toxic shock, is also present, along with other virulence determinants such as peptidases, ureases, lipases, and chitinases. Chemotaxis proteins were also found, suggesting that the isolate, which is motile, is capable of chemotaxis [42].

The detection of a strain such as *Paenibacillus* sp. strain VT 400 in patients with hematological malignancies is a critical result, especially in light of in vivo studies. In these experiments, mice intranasally challenged with at least 8.5 log_10_ CFU of the isolate died from pneumonia, and were found to have infected lungs as well as spleen, indicating dissemination of the infection. Taken together, the data suggest that the strain not only presents genetic features of pathogenic bacteria, but may indeed trigger a life-threatening infection.

In addition, the genome of *Paenibacillus* sp. strain VT 400 features numerous multidrug efflux transporters known to confer intrinsic and acquired resistance to many antibiotics used in clinical practice [43]. These proteins catalyze uptake, efflux, diffusion, solute exchange, and other mechanisms of bacterial defense against xenobiotics [44, 45]. In addition, these transporters are not drug-specific and are associated with multidrug resistance [46].

Moreover, the isolate contains two SMR efflux pumps, which are hallmarks of nosocomial infections and imply that *Paenibacillus* sp. strain VT 400 is most likely a circulating hospital strain, or a strain circulating among hematology patients [47]. SMR efflux pumps confer nosocomial antibiotic resistance and poor sensitivity to biocidal quaternary ammonium compounds [48, 49]. Notably, SMR proteins have never been previously found in *Paenibacillus*.

We detected chloramphenicol acetyltransferase, macrolide ABC transporter, vancomycin resistance protein, and *FosB*, which confer resistance to chloramphenicol, macrolide, vancomycin, and fosfomycin, respectively [50–52]. A bacteriocin resistance gene was also found, as were tetracycline resistance genes, including *TetA* [53, 54]. d-ala-d-ala ligase confers cycloserine resistance, while dihydrofolate reductase A is associated with resistance to trimethoprim and trimethoprim-sulfamethoxazole [55, 56]. In addition, the genome contains resistance genes to β-lactams, including metal-dependent hydrolases, as well as resistance genes to chemotherapeutic drugs.

Nevertheless, many resistance genes of *Paenibacillus* sp. strain VT 400 are not expressed, in accordance with the idea that many mutations do not lead to resistant phenotype [57]. Sporulation, such as in *Paenibacillus* sp. strain VT 400, preserves and disperses genetic material such as antibiotic resistance genes to overcome harsh environmental conditions [58, 59]. These spores may be particularly hazardous to immunocompromised patients.

## Conclusions

This study expands the number of poorly characterized *Paenibacillus* spp. that may cause pulmonary disease in humans [18]. We provide virulence and antibiotic resistance data based on draft genomes and antimicrobial susceptibility testing. We also demonstrate the ability of the strain to trigger pneumonia in vivo, and to invade spleen tissue. Our data may have important implications in the clinic, as the oral microbial flora in patients with hematological malignancies could be a reservoir of pneumonia-causing agents.

Whether *Paenibacillus* sp. strain VT 400 is more prevalent in individuals with acute leukemia remains to be established. However, it is clear that the isolate may have direct clinical implications for patients with therapy-induced immunosuppression. We now intend to determine the prevalence of *Paenibacillus* sp. strain VT 400 among different groups of patients, as well as among patients beyond hematology and bone marrow transplantation units.

## Availability of supporting data

The complete genome has been deposited in GenBank under the Accession No. LELF01000000. The type strain is deposited in the Deutsche Sammlung fur Mikroorganismen und Zellkulturen under Accession Number DSM 100755.
